# SOCIAL RISKS AMONG PREDOMINANTLY AFRICAN AMERICAN/BLACK CAREGIVERS OF PERSONS LIVING WITH DEMENTIA

**DOI:** 10.1093/geroni/igad104.2354

**Published:** 2023-12-21

**Authors:** Victoria Winslow, Christina Bernhardy, Soo Borson, Stacy Tessler Lindau, Jennifer Makelarski, Katherine Thompson

**Affiliations:** University of Chicago, Chicago, Illinois, United States; UChicago Medicine, Chicago, Illinois, United States; University of Southern California, Los Angeles, California, United States; University of Chicago, Chicago, Illinois, United States; University of Chicago, Chicago, Illinois, United States; University of Chicago, Chicago, Illinois, United States

## Abstract

Health-related social risk factors (HRSRs) are associated with poorer quality of life and higher caregiving burden and are more prevalent among women and in African American/Black communities due to structural inequities. Little is known about gender differences in HRSRs among dementia caregivers, especially AA/B dementia caregivers. Baseline data were obtained from 345 caregivers of PWD enrolled (17% at their own and 83% at their care recipient’s point of care) in a single-blind, randomized controlled trial of CommunityRx-Caregiver, a social care intervention delivered at the point of health care. Gender differences in HRSR rates (food, housing, safety, utilities, transportation, financial, and social support) were tested using chi-square tests. Most caregivers were women (78%) and non-Hispanic AA/B (80%). Female caregivers had significantly higher rates of having HRSRs (64% of women vs. 51% of men, p=0.04, 62% overall). Lack of social support (47% of women vs. 38% of men, 45% overall), financial strain (26% vs. 23%, 25% overall), and housing insecurity (23% vs. 14%, 21% overall) were also most prevalent among women. Thirty-one percent of women compared to 23% of men reported 2 or more HRSRs. Health-related social risks, especially lack of social support, were prevalent among women and men dementia caregivers in a predominantly AA/B community. By 2030, more than 9 million Americans and their families will be affected by dementia and dementia caregiving. Social care interventions should be designed to identify and support caregivers at the point of their own or their care recipient’s healthcare.

